# Collision-Free Transmissions in an IoT Monitoring Application Based on LoRaWAN

**DOI:** 10.3390/s20144053

**Published:** 2020-07-21

**Authors:** Rahim Haiahem, Pascale Minet, Selma Boumerdassi, Leila Azouz Saidane

**Affiliations:** 1 RAMSIS Team, CRISTAL Laboratory, 2010 Campus University, 2010 Manouba, Tunisia; haiahem.rahim@gmail.com (R.H.); leila.saidane@ensi-uma.tn (L.A.S.); 2 EVA Project, Inria—Paris, 75012 Paris, France; 3 CEDRIC/CNAM, 75003 Paris, France; selma.boumerdassi@cnam.fr

**Keywords:** IoT, LPWAN, LoRaWAN, monitoring application, reliability, scalability, collision-free, TDMA, FDMA, air pollution monitoring

## Abstract

With the Internet of Things (IoT), the number of monitoring applications deployed is considerably increasing, whatever the field considered: smart city, smart agriculture, environment monitoring, air pollution monitoring, to name a few. The LoRaWAN (Long Range Wide Area Network)architecture with its long range communication, its robustness to interference and its reduced energy consumption is an excellent candidate to support such applications. However, if the number of end devices is high, the reliability of LoRaWAN, measured by the Packet Delivery Ratio (PDR), becomes unacceptable due to an excessive number of collisions. In this paper, we propose two different families of solutions ensuring collision-free transmissions. The first family is TDMA (Time-Division Multiple Access)-based. All clusters transmit in sequence and up to six end devices with different spreading factors belonging to the same cluster are allowed to transmit in parallel. The second family is FDMA (Frequency Divsion Multiple Access)-based. All clusters transmit in parallel, each cluster on its own frequency. Within each cluster, all end devices transmit in sequence. Their performance are compared in terms of PDR, energy consumption by end device and maximum number of end devices supported. Simulation results corroborate the theoretical results and show the high efficiency of the solutions proposed.

## 1. Introduction

The Internet of Things (IoT) is tremendously contributing to the increase of the number of monitoring applications deployed. These applications belong to various fields like smart farming (with smart irrigation, nutrient monitoring and disease detection) [1,2], air pollution monitoring [3,4], smart cities [5] (with smart parking, smart waste collection and smart lighting), environment control (with leak detection), etc. Such applications require long range transmissions (e.g., from 1 to 12 km), a high number (i.e., ≥ 900) of End Devices (EDs) transmitting short messages (i.e., less than 50 bytes) to a sink in charge of gathering data, not frequently (e.g., from once every 5 mn, up to once every hour or every 6 h). The high number of EDs deployed leads to the choice of low-cost wireless transceivers. In addition, since these devices are battery-operated, a high battery lifetime is required. Taking into account the features of IoT monitoring applications, namely their monitoring area that ranges over an area of up to 6 km radius around the gateway, their data rate, the robustness against interferences and a high network lifetime requested, the LoRaWAN solution [4,6] is the most attracting solution, compared with other examples of Low-Power WANs (LPWANs) such as Sigfox and NB-IoT (Narrow Band—Internet of Things)which enable long communication range and operate for long periods [7].

However, many authors [8,9,10,11,12], observed that the reliability of LoRaWAN considerably decreases when the number of EDs increases: the Packet Delivery Ratio (PDR) drops by 50% for 900 EDs. This scalability problem is an issue for LoRaWAN. The objective of this paper is to get rid of the unreliability of LoRaWAN due to collisions when the number of EDs increases. For that purpose, we propose and compare different solutions based on LoRaWAN [13] which ensure collision-free transmissions for monitoring applications. The first solution, called OAPM_D for Orthogonal Air Pollution Monitoring Deterministic solution, is based on a time division multiple access (TDMA) of clusters, where a cluster is a group of EDs, whereas the second solution, called FAPM for FDMA-based Air Pollution Monitoring solution, is based on a frequency division multiple access (FDMA) of clusters. These basic solutions are optimized to support more End Devices (EDs) without losing messages and are compared on five different configurations, with uniform distribution of spreading factors or not. The evaluation criteria are: (i) the number of EDs supported, (ii) the reliability of the networking solution evaluated by the Packet Delivery Ratio, (iii) the energy consumed by each ED, (iv) the ED lifetime, (v) the time needed to collect the monitoring report of all EDs. To get quantitative performance results, an air pollution monitoring application is considered for an illustrative purpose, and the format of the air pollution monitoring report is given. More generally, the solutions proposed are valid for any IoT monitoring application where each ED has a monitoring report to transmit once per monitoring period. We also discuss the applicability of these solutions according to the application requirements in terms of latency, number of EDs deployed and monitoring area size.

This paper is organized as follows. In Section 2, we present some IoT monitoring applications in smart agriculture, smart cities and environment monitoring. The LoRaWAN architecture is described as well as some solutions dealing with the unreliability of LoRaWAN. Section 3 defines the framework of this study. OAPM-D, a TDMA-based solution, and its optimized variant OAPM-O are detailed in Section 4, whereas Section 5 deals with FAPM, a FDMA-based solution, and its optimized variant FAPM-O. These solutions are compared from a theoretical point of view and then by simulation. In Section 6, the air pollution monitoring application is used for an illustrative purpose to evaluate the performances of our solutions. Theoretical and simulation results confirm the very good reliability obtained for a high maximum number of EDs. Complexity, ED energy consumption, network lifetime and data gathering delay are evaluated. In Section 7, the applicability of these solutions is discussed with regard to the IoT application requirements. A hybrid solution minimizing the gathering delay is proposed in Section 8. Finally, Section 9 summarizes the main results and gives some perspectives.

## 2. Related Work

### 2.1. IoT Monitoring Applications

IoT monitoring applications are various. They are already deployed in smart agriculture, smart cities, environment monitoring, etc. In [1], the authors present a review of long-range (LoRa) and (LoRaWAN)-enabled IoT applications for smart agriculture. They analyze the currently available long-range wide-area network technologies that could be the most appropriate for agriculture and agri-tech applications. Since in the agri-tech context, End Devices (EDs) usually monitor and report environmental factors such as temperature, humidity, and chemical conditions of soil and plants that do not require up-to-date real-time monitoring, authors conclude that LoRaWAN technology is the most suitable competitor for this type of application. Thus, the paper discusses the limits and outstanding research limitations of LoRaWAN and points out the biggest challenge and issue of the future development of large-scale LoRaWAN applications: the effect of packet collision on the deployment scalability.

In [14], the authors develop an IoT agriculture system based on LoRaWAN. The system is made up of three services: the data collection service, the data analytics service and a remote control service. EDs transmit their measures to the LoRaWAN gateways (GWs) which transmit them to The Things Network (TTN) platform used to register EDs. GWs reroute the formatted messages to the cloud services. The cloud is in charge of data storage, data analytics and visualization. As a proof of concept, authors positioned three collector nodes (EDs) and one executor nodes in a 1-km radius from the GW. Air temperature, humidity and soil moisture measured by EDs are used by the analytics service to decide about the irrigation system. The decision is sent to the executor of the watering pump in the field, in order to turn it on or off, according to the decision received.

In [15], the authors present a long term evaluation of LoRaWAN for smart city IoT deployments. The paper details experiences from deploying a city-scale LoRaWAN network across Southampton, UK. The aims behind this deployment are to support an installation of air quality monitoring and to explore the capabilities of LoRaWAN protocol. Furthermore, the authors compare different LoRaWAN nodes and Gateways needed for the deployment. Based on the data-set produced over 135,000 transmissions gathered while monitoring air quality, the authors analyze message delivery reliability and delay, as well as the scheduling and the atmospheric influences on message delivery. Finally, authors assert that LoRaWAN is an applicable communication technology for city-scale air quality monitoring and other smart city applications.

In [16], the authors aim to demonstrate the feasibility of an Air Quality (AQ) monitoring system based on Internet of Things (IoT) devices. Each IoT device is equipped with several sensors able to sense the variations in time and space of Particulate Matter (PM) air pollutants. Power and network connectivity are provided by a Power Over Ethernet (PoE) HAT to a Raspberry Pi 3 Model B. Each IoT device results in a total cost of approximatively 900 USD (with four PM sensors supported per device) in its first version and approximatively 1000 USD (up to ten PM sensors supported per device) in the second one. To allow low bandwidth and long range communication, a LoRaWAN module has been included. Six IoT devices were deployed in two school sites in Southampton to monitor PM concentration over a period of seven months. Results show that on the one hand, the Spreading Factor (SF) 10 gives the best trade-off in terms of range and throughput. On the other hand, the capability of these IoT devices to sense spatio-temporal variations of air pollutants with a lower cost than this of an Automatic Urban and Rural Network (AURN) station is established.

In [3], the authors present a real-life 100-day deployment of low-cost black carbon (BC) sensors across 100 distinct sampling locations in the 15 km${}^{2}$ neighborhood in West Oakland, California. The residents, organizations and businesses were recruited to host Aerosol Black Carbon Detector (ABCD) units. Data from the sampling location is aggregated in an online database containing 1-minute average black carbon (BC) concentration measurements wirelessly transmitted by each ABCD and stored in a custom SQL database online. BC concentration measurements were collected over a period of 240,000 h and used to raise awareness of the harmful effects of air pollution on health.

### 2.2. LoRaWAN and Its Reliability Problem

As seen in the previous subsection, many authors agree on the fact that LoRaWAN meets many requirements of IoT monitoring applications. We now briefly present LoRaWAN, before pointing out its reliability problem and summarizing some solutions based on a time slotted medium access.

LoRa uses the chirp spread spectrum (CSS) modulation to provide a long-range communication link robust to interference [17]. According to the ERC Recommendation 70-03 [18], LoRa operates in the 868 MHz band in Europe and in the 915 MHz band in North America. Table 1 presents LoRaWAN default channels and duty cycle limitations for the 868 MHz band, where each channel has a bandwidth of 125 kHz. LoRa supports different transmission bitrates depending on the Spreading Factor (SF) used. The SF value ranges from 7 with the highest bitrate of 5470 bps and a distance up to 2 km, to 12 with the lowest bit rate of 250 bps and a distance up to 6 km in urban area [6]. The main advantage in this modulation is that several transmissions with different SFs on the same channel are orthogonal and can be received simultaneously by the GW, provided that they are allowed by the number of receive paths [19,20].

LoRaWAN™ [13] defines the architecture of a network based on LoRa. There are three main types of devices: Network Server (NS), Gateways (GWs) and End Devices (EDs). The EDs form a star topology around the GW. All EDs transmit their messages to the GW according to the Aloha medium access method. Three different classes of EDs exist, namely:

- Class A devices, with the basic set of features that all devices must implement. Hence, class A is the default class with the lowest power consumption class [7].
- Class B devices, in addition to the functionalities of Class A, can be accessed by the GW at some predefined time slots defined by Beacon messages periodically sent by the gateway [11].
- Class C devices are Continuously listening End Devices. Hence, they are accessible with low-latency but consume more energy than EDs of any other class [10].

The complexity GW forwards the messages received from the EDs to the cloud-based Network Server via standard IP connections. Because of its multi-channel transceiver, the GW is able to simultaneously receive several messages on multiple channels (i.e., up to eight channels).

The complexity is kept within the NS [13] with the elimination of redundant received packets, security checks, acknowledgment and downlink traffic scheduling, data rate adaptation, etc.

Many authors, [12,21,22,23], observed that when the number of End Devices (EDs) increases, the Packet Delivery Ratio (PDR) of LoRaWAN falls below the ratio acceptable by the application. This is due to the high number of collisions arising with the Aloha medium access method. In [23], the authors study the performance of LoRaWAN especially to find network capacity and understand how transmission reliability depends on the number of packets generated by the end devices. Assuming that the messages are generated according to a Poisson distribution, the comparison of the developed mathematical model and the simulation reveals that the collisions resolution approach used in LoRaWAN is inefficient with a high number of EDs. In order to limit the collision, the authors show with simulation that when the transmission frequency is 10 Hz and the biggest payload size of the greatest SF used is 51 bytes, the communication is rather reliable (packet loss ratio less than 0.001) if the number of EDs is 100 and each ED sends at most one packet per 20 min. The network capacity may reach 5000 EDs when each of them generates two messages per day on average.

Our paper aims at proposing solutions to avoid collisions, whereas others try to strongly limit them [23] or transform them into constructive collisions like Choir [24] or QuAiL [25] using the linear addition of powers of phase-asynchronous channels. Others take advantage of specific hardware to successfully decode weak transmissions at the GW, like Charm [26].

### 2.3. Solutions Based on Time Slots

Several solutions to improve the reliability of LoRaWAN, such as [8,9,27], rely on a time slotted medium access. In [27], the authors propose to regulate the medium access within slots (Slotted Aloha). For this purpose, they introduce a time synchronization service for low-cost IoT LoRaWAN devices connected to a gateway. When any ED transmits a confirmed uplink frame, it includes its timestamp at the end of its transmission. The GW sends a timestamped Acknowledgement in the RX1 receive window. Thus, the ED has all the information needed for clock re-alignment. The authors developed and deployed a LoRaWAN testbed in real-life conditions. They show that their solution improves the reliability in real-life deployments based on LoRaWAN.

In [8], each End Device is assigned a time slot, whose value is computed from its own address and the frame length. The last slot of the frame is used by the GW on the one hand, to synchronize the EDs and on the other hand, to group the acknowledgments of transmissions sent in this frame. The frame length dynamically evolves over time to reflect the real number of EDs in the LoRaWAN network.

In [9], time slots are assigned to End Devices (EDs) according to their traffic needs. The synchronization and scheduling methods are triggered by any ED to get its time slot indexes encoded in Bloom filters to reduce the size of scheduling messages. They show that this solution improves the reliability of LoRaWAN. However, the scalability problem remains for a large number of devices, or even a moderate number of devices but with high SFs.

## 3. Framework for Performance Evaluation

### 3.1. Notations

In this paper, we adopt the notations given in Table 2.

### 3.2. General Architecture

**Figure 1:**
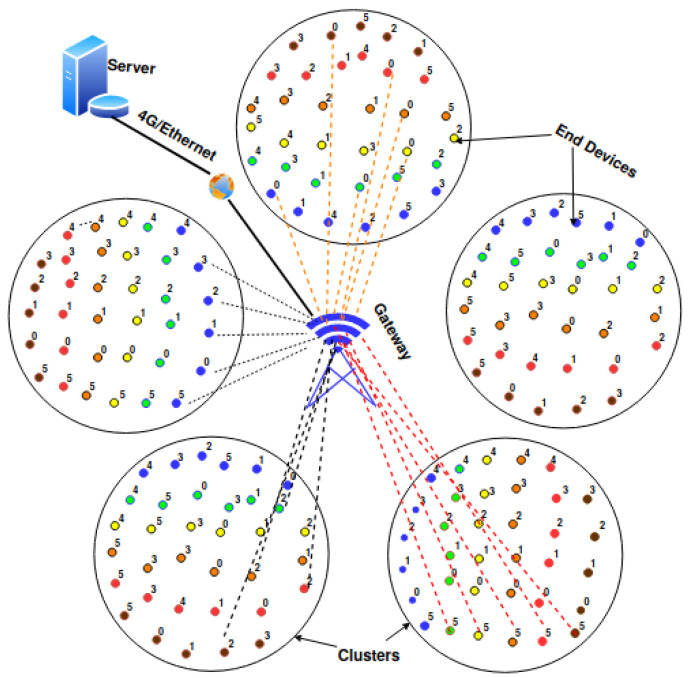
General network architecture with 5 clusters in the monitoring area.

An example of LoRaWAN architecture is depicted in Figure 1. The monitoring area consists of five clusters represented by the five disks and populated by End Devices (EDs). Each ED is represented by a small dot whose color refers to the Spreading Factor (SF) used by this ED. The meaning of the number associated with the ED in Figure 1 depends on the solution proposed. For instance, it can be the identifier of the ED sub-cluster, or the frequency used by this ED.

More generally, the architecture for the LoRaWAN network supporting monitoring applications can be described as follows:

A1 A single gateway (GW) exists in the monitoring area. It is located in the center of the monitoring area to reduce its distance to EDs, whose activity is ruled by their duty cycle limitation (i.e., 1%).
A2 This gateway has a number of frequency channels F=3, 6 or 8. Three is the default number, whereas eight is the maximum number of frequency channels that a commercially available GW can listen to, see for instance the technical features of the SX1301 digital baseband chip [6].
A3 The gateway can simultaneously demodulate several messages using different spreading factors even on the same frequency channel. However, the gateway cannot demodulate more than M=8 messages simultaneously [6]. *M* is also called the number of receive paths in the literature.
A4 The monitoring area is split into angular sectors centered at the gateway as depicted in Figure 1. Each angular sector is called a cluster. Clusters are populated by End Devices (EDs), according to their geographical coordinates obtained when the EDs are deployed.
A5 All EDs are LoRaWAN [13] class A devices, which is the basic class and the most energy-efficient one.
A6 All EDs are one-hop away from the GW. In other words, the network topology is a star centered at the GW.
A7 Each ED uses a spreading factor SF∈{SF7,SF8,SF9,SF10,SF11,SF12} that depends on its distance to the GW.
A8 Each ED transmits a single message per monitoring period, denoted MP. This message contains its monitoring report. This uplink traffic is not acknowledged (i.e., unconfirmed data type).
A9 Each ED transmits its monitoring message at a time and on a frequency channel assigned by the Network Server according to the solution considered (e.g., Algorithm 1 for FAPM in Section 5).
A10 All EDs are synchronized with regard to the reference time of the GW. All EDs are kept synchronized within Δ from the GW. The synchronization period is denoted SP.

It is worth noting that all the solutions proposed in this paper rely on Transmission Times assigned by the Network Server to EDs to ensure collision-free transmissions. However, this is not sufficient to avoid overlapping transmissions of two successive transmissions made by two different EDs, since the clocks of EDs drift apart over time. To get rid of this problem, EDs are periodically synchronized to the reference time provided by the GW and two guard periods are introduced:

- SG the synchronization guard to avoid either the overlapping of either a previous uplink transmission and the downlink synchronization message, or the overlapping of a previous downlink synchronization message and an uplink transmission.
  (1) SG=Δ+max_propagation_delay
- MG the monitoring guard to avoid overlapping of two successive uplink transmissions made by two different EDs.
  (2) MG=2Δ+max_propagation_delay

Several synchronization algorithms exist. Some adopt an on-demand approach, where each ED transmits its synchronization request to the GW. To reduce the energy consumption, simple algorithms synchronizing all the EDs in one-shot are preferred such as [28,29].

**Figure 2:**
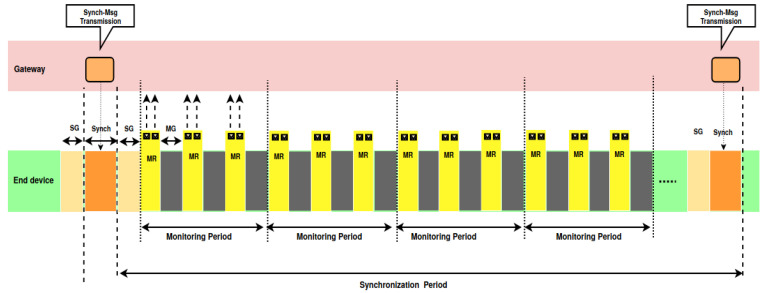
Medium activity over time.

Hence, the medium activity over time is organized into synchronization periods, as depicted in Figure 2, where Sync denotes the synchronization message and MR the monitoring report message.

Each synchronization period is delimited by the transmission of two successive synchronization messages. After the transmission of the synchronization message, several monitoring periods follow. A monitoring period consists of several Time Windows. Each cluster in the monitoring area is assigned a Time Window and one or several frequencies to transmit the monitoring reports of its EDs. Each ED is assigned a Transmission Time within the Time Window of its cluster and one frequency among those assigned to its cluster. Those assignments are such that all transmissions are collision-free. As a consequence, the following two constraints should be met:

*Constraint C1:*  The synchronization period contains nMPperSP monitoring periods with nMPperSP≥1:
  (3) $$T_{Sync}+2SG+nMPperSP\times MP\leq SP,$$
  where $T_{Sync}$ is the Transmission on Air of the synchronization message.
*Constraint C2:*  The monitoring period allows each ED to transmit its monitoring report without collisions. The expression of this constraint depends on the solution adopted (e.g., OAPM_D, FAPM, etc.).

### 3.3. Performance Evaluation Criteria and Additional Assumptions

In the comparative performance evaluation, we focus more particularly on the air pollution monitoring application. The air pollution level is evaluated by measuring the Air Quality Index (AQI) according to the recommendations given by the World Health Organization [30] and the US Environmental Protection Agency [31]. The AQI has six levels of air pollution with different impacts on the environment and people; these levels are Good, Moderate, Unhealthy for sensitive groups, Very Unhealthy and Hazardous. Six major pollutants have been identified as the causes of air pollution: Carbon Monoxide CO, Nitrogen Dioxide $NO_{2}$, Sulfur Dioxide $SO_{2}$, Ozone $O_{3}$, Particulate Matter $PM_{2.5}$ and Particulate Matter $PM_{10}$. The concentration of pollutant is averaged over one hour for $O_{3}$, $SO_{2}$ and $NO_{2}$, and over 24 h for Particulate Matter $PM_{2.5}$ and $PM_{10}$.

For the performance evaluation, we adopt some additional assumptions, which are not required by OAPM_D and FAPM, but make the performance computation easier (e.g., A11, A12), or allow a quantitative evaluation of the maximum number of EDs supported (e.g., A13 and A14):

A11 All clusters have the same distribution of SFs.
A12 The synchronization message is broadcast with the maximum SF used in the configuration considered. It has a total size of 17 bytes, where 4 bytes are used for the GW timestamp.
A13 The synchronization period is set to 1602 s, corresponding to a maximum propagation delay of 18 μs [32] associated with a radius of 6  km around the GW, SG=1.018 ms, MG=2.018 ms and Δ=1 ms. The monitoring period varies from 400 s to 1600 s.
A14 The monitoring report transmitted by each End Device (ED) has a message size of 21 bytes. In the monitoring message, each index of the six main air pollutants is coded on 10 bits, leading to 8 bytes of payload and a total of 21 bytes for the Monitoring message.

The deployment of the GW has to take into account multiple constraints: the real topography, the urban buildings and health rules, to name a few. This may result in various configurations where the GW is close to its EDs, or on the contrary, far from its EDs. To have a quantitative performance evaluation representative of various real-world deployments, we evaluate the LoRaWAN-based solutions proposed on five configurations, which are:

- $C_{16,16,16,16,16,16}$ A uniform configuration where the distribution of EDs within the monitoring area is uniform with regard to the different spreading factors. Each SF in {7,8,9,10,11,12} is used by 100/6 = 16.66% of the EDs in the monitoring area. Intuitively, this configuration has the same number of EDs in each concentric crown of width *R* around the GW, where *R* is the radio range of the smallest spreading factor. This means that the number of EDs with high spreading factors per unit of surface is reduced compared to the number of EDs with small spreading factors. It corresponds to a constant distribution of SFs.
- $C_{10,20,20,20,20,10}$ A non-uniform distribution where the minimum and the maximum SFs (i.e., SF7 and SF12) are used by 10% of the EDs, whereas the other SFs are used by 20% of the EDs. This configuration is close to the uniform one, except that the number of EDs very close and the number of EDs very far are smaller. This corresponds to a one-step distribution of SFs.
- $C_{33,33,33,0,0,0}$ A non-uniform distribution where the only SFs present in the monitoring area are SF7, SF8 and SF9, in the same ratio. This configuration corresponds to a “best case” deployment, where all EDs are close to the GW.
- $C_{0,0,0,33,33,33}$ A non-uniform distribution where the only SFs present in the monitoring area are SF10, SF11 and SF12, in the same ratio. This configuration corresponds to a “worst case” deployment, where all EDs are far from the GW, which could not be installed closer to the EDs for diverse reasons.
- $C_{5,15,35,30,10,5}$ a non-uniform distribution where all SFs are present but with different percentages. SF7 is used by 5% of EDs, SF8 by 15%, SF9 by 35%, SF10 by 30%, SF11 by 10% and SF12 by 5%. This configuration corresponds to a deployment, where some EDs are very close to the GW whereas others are very far, but most of them are at a medium distance from the GW. The SF distribution shape is closer to a bell.

For each configuration, we evaluate the maximum number of EDs supported by OAPM_D, FAPM and their optimized variants, both by theoretical computation and by simulation. For theoretical results, we use the assumption A11 (i.e., all clusters have the same distribution as the monitoring area) to define the concept of representative of the ED distribution in the different SFs. A representative is a set of EDs with their associated SFs, such that each cluster contains a multiple of representatives. We evaluate the transmission time of a representative with each solution to deduce the maximum number of EDs per cluster.

## 4. OAPM_D, A TDMA-Based Solution

### 4.1. Presentation of OAPM_D

**Figure 3:**
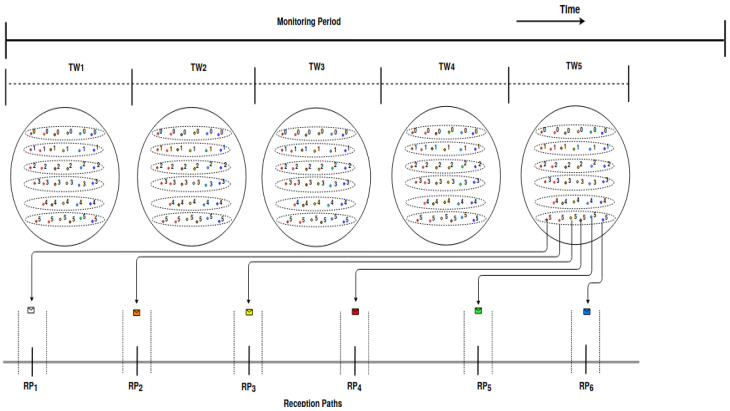
Time diagram of transmissions with OAPM_D.

In OAPM_D, clusters are subdivided into sub-clusters and each cluster transmits sequentially, similarly for the sub-clusters. The parallelism of transmissions is obtained within each sub-cluster where a number of EDs equal to the number of different SFs in this sub-cluster, transmits in parallel. In any case, each sub-cluster includes at most six EDs, which is the maximum number of different SFs present in this sub-cluster. OAPM_D is a deterministic solution where each ED within a sub-cluster transmits on a given frequency channel and at a transmission time given by the network server. The scheme of transmissions with OAPM_D is depicted in Figure 3, where the clusters are represented by disks, sub-clusters by dashed ovals and EDs by dots whose color represents their spreading factor. Each cluster *i* transmits sequentially in the time window $TW_{i}$ assigned to it. Similarly, the sub-clusters of cluster *i* transmit sequentially in $TW_{i}$. All the EDs belonging to the same cluster (i.e., at most six EDs) have different SFs and transmit simultaneously, which is illustrated by the six receive paths $RP_{k}$ at the bottom of Figure 3, where $RP_{k}$ denotes the *k*th receive path of the GW.

In OAPM_D, the choice of the frequency channel used by any ED is assigned by the Network Server, in such a way that at any time, there is at most one ED transmitting its message on a given frequency channel using a given SF. For OAPM_D, Constraint C2 leads to:

(4) $$\sum_{h=1}^{nsub}\left(T_{h}+MG\right)\leq MP$$

where nsub denotes the number of sub-clusters in the monitoring area and $T_{h}$ is the maximum transmission time on the air of a monitoring report with the maximum spreading factor in sub-cluster *h*.

Notice that in specific configurations, where some receive paths of the GW would remain unused because some spreading factors are missing in the cluster considered, some optimizations are possible. For instance in configurations $C_{33,33,33,0,0,0}$ and $C_{0,0,0,33,33,33}$, two EDs using the same SF could be grouped into the same sub-cluster provided that they are assigned two different frequency channels (see Section 4.3.3 and Section 4.3.4 for more details). This is the intuitive idea behind OAPM_O.

### 4.2. Presentation of OAPM_O

OAPM_O is the optimized version of OAPM_D, where the main principles of OAPM_D are kept: clusters and sub-clusters transmit sequentially, but EDs in the same sub-cluster transmit in parallel on the frequency channel and with the SF assigned by the Network Server. The definition of sub-cluster is generalized to allow the coexistence of several EDs using the same SF in the same sub-cluster, provided that they transmit on different frequency channels. This condition is required to ensure collision-free transmissions.

Notice that GW cannot listen to more than *F* channels with 3≤F≤8 and can demodulate at most M=8 messages simultaneously [6]. Hence, in OAPM_O, the number of EDs per sub-cluster may be higher than 6 which is the maximum allowed in OAPM_D. As a consequence, the number of EDs supported by OAPM_O is greater than or equal to that supported by OAPM_D, as we will see in the next section.

### 4.3. Theoretical Performances of OAPM_D and OAPM_O

#### 4.3.1. Configuration $C_{16,16,16,16,16,16}$

In the $C_{16,16,16,16,16,16}$ configuration, each cluster contains the same number of EDs for each SF in [7,12]. Hence, the representative is SF7,SF8,SF9,SF10,SF11,SF12. Since *F* the number of GW receive paths is at least equal to 6, each sub-cluster in the OAPM family contains exactly six EDs with six different SFs to guarantee collision-free transmissions. The number of sub-clusters in a cluster is limited by: $\left\lfloor \frac{MP}{T_{12}+MG}\right\rfloor$. Hence the maximum number of EDs supported by OAPM_D and OAPM_O is given by:

(5) $$ED_{OAPMD}=ED_{OAPMO}=6\times \left\lfloor \frac{MP}{T_{12}+MG}\right\rfloor ,$$

where $T_{12}$ denotes the Time on Air of the monitoring report using SF12.

#### 4.3.2. Configuration $C_{10,20,20,20,20,10}$

In the $C_{10,20,20,20,20,10}$ configuration, each cluster contains the same number of EDs for each SF in [8,11] and this number is twice the number of EDs with SF7, which is also the number of EDs with SF12. As a consequence, a representative of the cluster distribution is given by: SF7,SF8,SF9,SF10,SF11,SF12,SF8,SF9,SF10,SF11. The number of representatives in the monitoring area should be such that each ED is able to transmit once per monitoring period without a collision. Since by definition of OAPM_D, EDs with SF7 to SF12 can transmit in parallel (one ED per SF), the time needed by a representative to transmit is equal to $T_{12}+T_{11}+2MG$. Hence, the maximum number of representatives is given by $\left\lfloor \frac{MP}{T_{12}+T_{11}+2MG}\right\rfloor$. Since, each representative contains 10 EDs, the maximum number of EDs supported by OAPM_D and OAPM_O is given by:

(6) $$ED_{OAPMD}=ED_{OAPMO}=10\times \left\lfloor \frac{MP}{T_{12}+T_{11}+2MG}\right\rfloor .$$

#### 4.3.3. Configuration $C_{33,33,33,0,0,0}$

In the $C_{33,33,33,0,0,0}$ configuration, each cluster contains only EDs with SF in {7,8,9} and has the same number of EDs for each SF in {7,8,9}. Hence, a representative of cluster distribution is given by SF7,SF8,SF9. However, when at least two frequency channels are available, OAPM_O uses two frequency channels per sub-cluster allowing two EDs with the same SF to transmit simultaneously, but on two different channel frequencies. Hence with this improvement, a cluster representative, which is also a sub-cluster representative, consists of two EDS using SF7, two EDs using SF8, and two EDs using SF9. One ED with SF7, one ED of SF8 and one ED of SF9 transmit in parallel on a given channel, where the three others EDs of this sub-cluster transmit in parallel on another given channel. The transmission time required by a representative to transmit its message is given by $T_{9}+MG$. Hence, the maximum number of cluster representatives is given by $\left\lfloor \frac{MP}{T_{9}+MG}\right\rfloor$. Since, each representative contains six EDs, the maximum number of EDs supported by OAPM_O is given by:

(7) $$ED_{OAPMO}=6\times \left\lfloor \frac{MP}{T_{9}+MG}\right\rfloor .$$

whereas the number of EDs supported by OAPM_D is only

(8) $$ED_{OAPMD}=3\times \left\lfloor \frac{MP}{T_{9}+MG}\right\rfloor .$$

#### 4.3.4. Configuration $C_{0,0,0,33,33,33}$

The case of the $C_{0,0,0,33,33,33}$ configuration is very similar to the previous one, leading to the following maximum number of EDs supported by OAPM_O:

(9) $$ED_{OAPMO}=6\times \left\lfloor \frac{MP}{T_{12}+MG}\right\rfloor .$$

whereas the number of EDs supported by OAPM_D is only

(10) $$ED_{OAPMD}=3\times \left\lfloor \frac{MP}{T_{12}+MG}\right\rfloor .$$

#### 4.3.5. Configuration $C_{5,15,35,30,10,5}$

In the $C_{5,15,35,30,10,5}$ configuration, a cluster distribution representative is given by SF7,3SF8,7SF9,6SF10,2SF11,SF12, leading to one sub-cluster with SF7…SF12, one sub-cluster with SF8…SF11, one sub-cluster with SF8…SF10, three sub-clusters with SF9,SF10 and one sub-cluster with SF9. These sub-clusters have a total transmission time equal to $T_{12}+T_{11}+4\times T_{10}+T_{9}+7MG$. Hence, the maximum number of representatives per cluster is given by $\left\lfloor \frac{MP}{T_{12}+T_{11}+4\times T_{10}+T_{9}+7MG}\right\rfloor$. Since, each representative contains 20 EDs, the maximum number of EDs supported by OAPM_D is given by:

(11) $$ED_{OAPMD}=20\times \left\lfloor \frac{MP}{T_{12}+T_{11}+4\times T_{10}+T_{9}+7MG}\right\rfloor .$$

With OAPM_O, the idea consists in filling each sub-cluster with *M* EDs, while ensuring that at most *F* frequency channels and at most *M* receive paths are used. The sub-cluster membership is given in Table 3, where members of any sub-cluster are denoted in black, blue or red, depending on the frequency channel assigned to transmit the ED monitoring reports (i.e., a frequency channel is represented by a color: black, blue or red). We notice that increasing *F* from 3 to 6 or 8 does not change the membership of clusters, since each sub-cluster 1 or 2 already transmits *M* messages in parallel and consumes all the available receive paths of the GW.

(12) $$ED_{OAPMO}=20\times \left\lfloor \frac{MP}{T_{12}+T_{11}+T_{10}+3MG}\right\rfloor .$$

### 4.4. Discussion

Note that in OAPM_D, if the number of frequency channels of the GW is strictly higher than the maximum number of different SFs present in the sub-cluster, some frequency channels remain unused. To increase the maximum number of EDs supported, there are several possibilities. The first one has already been presented, it consists in optimizing the definition of sub-clusters by allowing two EDs with the same SF to coexist, provided that they use two different frequency channels. Another possibility consists in assigning different frequency channels to clusters, enabling them to transmit in parallel. This is the basic idea of FAPM presented in the next section.

## 5. FAPM, A FDMA-Based Solution

### 5.1. Description of FAPM

**Figure 4:**
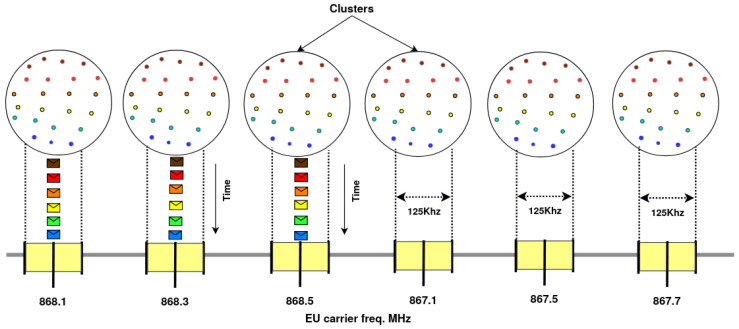
Transmissions with FAPM.

In FAPM, all clusters transmit in parallel, but on different frequency channels, and all EDs of a same cluster transmit sequentially as depicted in Figure 4. In FAPM, the Transmission Window associated with each cluster is equal to the whole monitoring period, unlike OAPM_D and OAPM_O where each cluster is assigned a part of the monitoring period. As depicted in Figure 4, each cluster is assigned its own frequency channel (i.e., a total of six frequencies assigned to the LoRaWAN network in this Figure). Within each cluster, all its EDs transmit sequentially.

Table 4 highlights the differences between OAPM_D and FAPM, as well as between their optimized variants. The last line of this table gives the maximum number of parallel transmissions in a same sub-cluster. This number is limited by:

- the number of receive paths assigned to the containing cluster, for all the four solutions. This number is reduced to one for FAPM.
- the number of different SFs present in this sub-cluster, for OAPM_D and FAPM_O.
- the number of different SFs present in this sub-cluster times the number of frequencies assigned to this sub-cluster, for OAPM_O.

In this table, ‘# of RPs’ means the number of receive paths assigned to the containing cluster.

‘# of ≠ SFs in this sub-cluster’ denotes the number of different SFs present in this sub-cluster.

‘# of ≠ SFs in the sub-cluster × # of Freq assigned to this sub-cluster’ means the number of different SFs present in this sub-cluster multiplied by the number of frequencies assigned to this sub-cluster (see the last line for OAPM_O in Table 4).

As a consequence, the maximum number of clusters in FAPM is equal to the minimum between *F* the number of frequency channels of the GW, and *M* the number of messages the GW is able to demodulate simultaneously. For FAPM, Constraint C2 can be interpreted as: each cluster must end the transmissions of all its EDs by the end of the monitoring period, which can be written as:

(13) $$max_{i}\left(\sum_{h=1}^{nED_{i}}\left(T_{h}+MG\right)\right)\leq MP$$

where *i* is the cluster index, $nED_{i}$ is the number of EDs in cluster *i* and $T_{h}$ is the transmission time in the air of a monitoring report with the spreading factor used by ED *h* in cluster *i*.

### 5.2. System Behavior with FAPM

The system behavior with FAPM consists of an initialization phase during which the Network Server configures all EDs (see Algorithm 1), followed by the synchronization phase and one or several monitoring phases. Then a new periodic synchronization phase is entered and so on (see Algorithm 2). To reduce the bandwidth and processing overhead, the synchronization period is maximized and a compensation is computed by each ED before transmitting or receiving. This computation is done assuming a linear clock drift within the synchronization period.
**Algorithm 1** End Devices Configuration (Run by the server to configure all End Devices)/* Initializations */**for** each ED in the monitored area **do** /* Cluster assignment */
 Assign ED to a cluster according to its geographic coordinates obtained during deployment Compute minSF and maxSF the minimum and maximum SF used in the network**end for**/* Compute the parameters common to all EDs */Initialize SP, MP, SP_Start, $MP1\leftarrow SG+T_{Sync}$, nMPperSP, maxSF/* Compute the transmission and receive times of all EDs */**for** each RP=1 to *M* **do** I(RP)←1 /* Initialize the transmission index per receive path */**end for****for** each cluster in the monitored area **do** **for** each ED∈cluster **do**  /* Assign a receive path to each ED and an index on this receive path */
  ED.RP← a receive path ∈[1,M]  Index←I(RP)
  I(RP)++
  /* Assign Transmission Time to this ED */
  $ED.TT\leftarrow \sum_{k=1}^{Index-1}T_{k}+\left(Index-1\right)MG$ /* From the beginning of the monitoring period */ **end for** /* ED */
**end for**/* Cluster *//* Send configuration parameters to all EDs */**for**SF=minSF to maxSF **do** Unconfigured← all the EDs using SF **while** Unconfigured≠empty **do**  Multicast configuration parameters to a maximum number of EDs ∈Unconfigured using SF  Remove these EDs from Unconfigured **end while****end for**

**Algorithm 2** Monitoring step (Run by any End Device ED)
Receive (ConfigurationParameters)Initialize its local parameters
NextAwake←SP_Start
NbS=0 /* Number of the current synchronization period */
**repeat**
 /* Behavior of any ED during a synchronization period */ Sleep until NextAwake to receive the next synchronization message Process the synchronization message, Update the clock Comp← the compensation before next transmit NbS++
 NextAwake←SP_Start+MP1+ED.TT+Comp
 **for** NbM=1 to nMPperSP **do**  /* Transmit its monitoring report once per MP  during nMPperSP successive periods */  Sleep until NextAwake to transmit its monitoring msg  Build the air pollutant report  Transmit the air pollutant report to the GW  Comp← the compensation before next transmit  NextAwake←SP_Start+MP1+NbM*MP+ED.TT+Comp
 **end for** Comp← the compensation before next receipt NextAwake←SP_Start+NbS*SP+Comp
**until** forever


### 5.3. Presentation of FAPM_O

In FAPM, the maximum number of clusters is upper limited by *F* the number of frequency channels of the gateway and by *M* the maximum number of messages demodulated simultaneously. It is equal to min(F,M). The maximum number of EDs per cluster is limited by the fact that all the EDs of a same cluster should transmit sequentially in a time less than or equal to the monitoring period. However, for all configurations where the number of frequency channels of GW is strictly less than *M* the maximum number of messages that can be demodulated simultaneously, FAPM is not optimal. For this reason, FAPM_O is introduced to improve the performances of FAPM when M>F by allowing up to *M* messages to be simultaneously demodulated in some clusters.

For FAPM_O, Constraint C2 can be written as:

(14) $$max_{i}\left(\sum_{h=1}^{nsub_{i}}\left(T_{h}+MG\right)\right)\leq MP,$$

where *i* denotes the cluster index, $nsub_{i}$ denotes the number of sub-clusters in cluster *i* and $T_{h}$ is the maximum transmission time in the air of a monitoring report with the spreading factor used by any ED in sub-cluster *h* of cluster *i*.

It follows that the number of EDs supported by FAPM increases with the number of frequency channels of the GW, as long as F≤M. When M>F, FAPM_O improves FAPM by demodulating simultaneously several messages per cluster.

### 5.4. Theoretical Performances of FAPM and FAPM_O

In this section, the maximum numbers of EDs supported by FAPM and its optimized variant FAPM_O, respectively, are evaluated.

#### 5.4.1. Configuration $C_{16,16,16,16,16,16}$

In the $C_{16,16,16,16,16,16}$ configuration, each cluster contains the same number of EDs per SF in [7,12]. The number of clusters is limited by *F* the number of GW frequency channels. In each cluster, all the transmissions are done sequentially. A cluster representative is given by one ED per SF, leading to six EDs per cluster representative. The transmission time needed by a cluster representative is equal to $\sum_{i=7}^{12}\left(T_{i}+MG\right)$. Hence to guarantee collision-free transmissions, the number of EDs per cluster is limited by: $6\left\lfloor \frac{MP}{\sum_{i=7}^{12}\left(T_{i}+MG\right)}\right\rfloor$. Hence, the maximum number of EDs supported by FAPM is given by:

(15) $$ED_{FAPM}=6F\times \left\lfloor \frac{MP}{\sum_{i=7}^{12}\left(T_{i}+MG\right)}\right\rfloor .$$

FAPM_O has the same behavior as FAPM when M≤F. For M=8 and F=3, FAPM_O allows transmission parallelism between $\lfloor \frac{M}{F}\rfloor =2$ EDs in the same cluster and with different SFs. As a consequence, FAPM_O allows in each cluster one ED of SF12 with one ED of SF11 to transmit in parallel, then one ED of SF10 and one ED with SF9, and finally one ED with SF8 with one ED of SF7. This is obtained by using two receive paths per cluster. This can be represented by:
ED1SF12SF10SF8ED2SF11SF9SF7Time$T_{12}$$T_{10}$$T_{8}$

In this case, the maximum number of EDs supported by FAPM_O is given by:

(16) $$ED_{FAPMO}=3*6\times \left\lfloor \frac{MP}{\left(T_{12}+T_{10}+T_{8}+3MG\right)}\right\rfloor .$$

#### 5.4.2. Configuration $C_{10,20,20,20,20,10}$

In the $C_{10,20,20,20,20,10}$ configuration, the number of EDs with SF = 7 and the number of EDs with SF12 represent 10% of the total number of EDs, whereas the number of EDs with any other SF represents 20% of the total number of EDs. In such a case, each ED pattern SF7,SF8,SF9,SF10,SF11,SF12,SF8,SF9,SF10,SF11 needs a time equal to $\sum_{i=7}^{12}\left(T_{i}+MG\right)+\sum_{i=8}^{11}\left(T_{i}+MG\right)$ to transmit the pollutant reports. Hence the maximum number of EDs per cluster is given by $10\times \left\lfloor \frac{MP}{\sum_{i=7}^{12}\left(T_{i}+MG\right)+\sum_{i=8}^{11}\left(T_{i}+MG\right)}\right\rfloor$. Since there is one cluster per frequency channel of the GW, the maximum number of EDs supported by FAPM is given by:

(17) $$ED_{FAPM}=10F\times \left\lfloor \frac{MP}{\sum_{i=7}^{12}\left(T_{i}+MG\right)+\sum_{i=8}^{11}\left(T_{i}+MG\right)}\right\rfloor .$$

FAPM_O has the same behavior as FAPM when M≤F. For M=8 and F=3, FAPM_O allows in each cluster two messages to be simultaneously transmitted as follows:
ED1SF7SF8SF9SF10SF11ED2SF8SF9SF10SF11SF12Time$T_{8}$$T_{9}$$T_{10}$$T_{11}$$T_{12}$

In this case, the maximum number of EDs supported by FAPM_O is given by:

(18) $$ED_{FAPMO}=3*10\times \left\lfloor \frac{MP}{\sum_{i=8}^{12}\left(T_{i}+MG\right)}\right\rfloor .$$

#### 5.4.3. Configuration $C_{33,33,33,0,0,0}$

In the $C_{33,33,33,0,0,0}$ configuration, there are only EDs with SF = 7, 8 or 9 and the number of EDs is fairly distributed over these three SF values. The EDs of a same cluster transmit sequentially. Hence, it will take a time equal to $\sum_{i=7}^{9}\left(T_{i}+MG\right)$ for the EDs belonging to the pattern SF7,SF8,SF9 to transmit their report. As a consequence, the number of EDs per cluster is limited by $3\times \lfloor \frac{MP}{\sum_{i=7}^{9}\left(T_{i}+MG\right)}\rfloor$. Since there is one cluster per frequency channel of the GW, the maximum number of EDs supported by FAPM is given by:

(19) $$ED_{FAPM}=3F\times \left\lfloor \frac{MP}{\sum_{i=7}^{9}\left(T_{i}+MG\right)}\right\rfloor .$$

FAPM_O has the same behavior as FAPM when M≤F. For M=8 and F=3, FAPM_O allows in each cluster two messages to be simultaneously transmitted as follows:
ED1SF7SF8SF9ED2SF9SF7SF8Time$T_{9}$$T_{8}$$T_{9}$

In this case, the maximum number of EDs supported by FAPM_O is given by:

(20) $$ED_{FAPMO}=6*3\times \left\lfloor \frac{MP}{T_{8}+2*T_{9}+3*MG)}\right\rfloor .$$

#### 5.4.4. Configuration $C_{0,0,0,33,33,33}$

This case is very similar to the previous one, leading to the following maximum number of EDs for FAPM:

(21) $$ED_{FAPM}=3F\times \left\lfloor \frac{MP}{\sum_{i=10}^{12}\left(T_{i}+MG\right)}\right\rfloor .$$

FAPM_O has the same behavior as FAPM when M≤F. For M=8 and F=3, FAPM_O allows in each cluster two messages to be simultaneously transmitted in each cluster as follows:
ED1SF10SF11SF12ED2SF12SF10SF11Time$T_{12}$$T_{11}$$T_{12}$

In this case, FAPM_O supports the maximum number of EDs given by:

(22) $$ED_{FAPMO}=6*3\times \left\lfloor \frac{MP}{T_{11}+2*T_{12}+3*MG}\right\rfloor .$$

#### 5.4.5. Configuration $C_{5,15,30,35,10,5}$

In the $C_{5,15,35,30,10,5}$ configuration, a cluster distribution representative is given by SF7, 3SF8, 7SF9, 6SF10, 2SF11, SF12. Since in FAPM, all EDs belonging to the same cluster transmit sequentially, the total transmission time of a cluster representative is equal to $T_{12}+2\times T_{11}+6\times T_{10}+7\times T_{9}+3\times T_{8}+T_{9}=20MG$. Hence, the maximum number of representatives is given by $\left\lfloor \frac{MP}{T_{12}+2\times T_{11}+6\times T_{10}+7\times T_{9}+3\times T_{8}+T_{9}+20MG}\right\rfloor$. Since, each representative contains 20 EDs, and there are *F* clusters, the maximum number of EDs supported by FAPM is given by:

(23) $$ED_{FAPM}=20F\times \left\lfloor \frac{MP}{T_{12}+2\times T_{11}+6\times T_{10}+7\times T_{9}+3\times T_{8}+T_{7}+20MG}\right\rfloor .$$

FAPM_O has the same behavior as FAPM when M≤F. For M=8 and F=3, FAPM_O allows in each cluster two messages to be simultaneously transmitted in each cluster as follows:
ED1SF7SF9SF11SF8SF10SF8SF9SF9SF9SF9ED2SF8SF10SF12SF9SF11SF9SF10SF10SF10SF10Time$T_{8}$$T_{10}$$T_{12}$$T_{9}$$T_{11}$$T_{9}$$T_{10}$$T_{10}$$T_{10}$$T_{10}$

In this case, FAPM_O supports the maximum number of EDs given by:

(24) $$ED_{FAPMO}=20*3\times \left\lfloor \frac{MP}{T_{12}+T_{11}+5*T_{10}+2*T_{9}+T_{8}+10*MG}\right\rfloor .$$

## 6. Comparative Performance Evaluation

In this section, we first compare the theoretical results of OAPM_D, OAPM_O, FAPM and FAPM_O, and then show by simulation that the solutions studied truly support this maximum number of EDs by providing them a Packet Delivery Ratio equal to one.

### 6.1. Comparison of Theoretical Results

**Figure 5:**
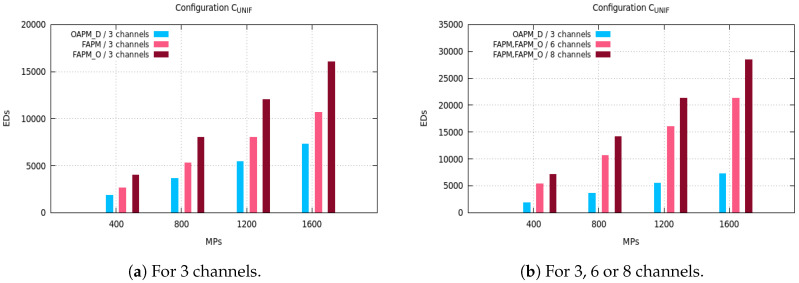
Number of EDs supported by OAPM_D, FAPM and FAPM_O in $C_{16,16,16,16,16,16}$.

**Figure 6:**
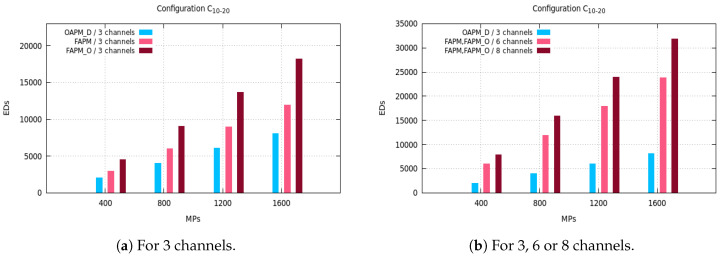
Number of EDs supported by OAPM_D, FAPM and FAPM_O in $C_{10,20,20,20,20,10}$.

**Figure 7:**
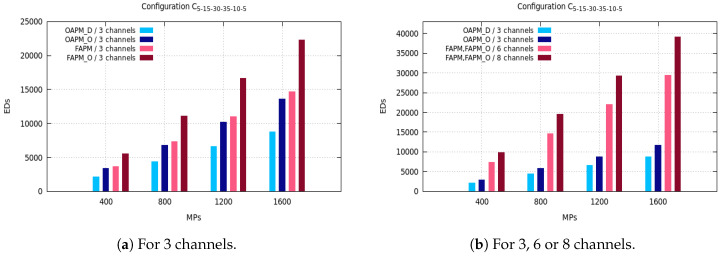
Number of EDs supported by OAPM_D, OAPM_O, FAPM and FAPM_O in $C_{5,15,30,35,10,5}$.

The maximum number of EDs supported by OAPM_D, OAPM_O, FAPM and FAPM_O are compared for various values of the monitoring period and various numbers of frequency channels on the five configurations described in Section 3.3. Figure 5, Figure 6 and Figure 7 depict these numbers of EDs for a monitoring period ranging from 400 s to 1600 s, and a number of frequency channels equal to 3, 6 or 8.

In Figure 5a, we see that the number of EDs supported per monitoring period in OPAM_D when exploiting six reception paths of the GW is always lower than that number in FAPM and FAPM_O using three and six channels, respectively. This is explained by the fact that each cluster in OAPM_D occupies six reception paths for the whole TW duration, whereas in FAPM a cluster does not occupy more than one reception path in the same duration. Since all SFs are present in each sub-cluster, six receptions paths are used, no optimization can be made for OAPM_D. However, when *F* the number of channels is lower than *M* the number of messages simultaneously demodulated, FAPM_O allows $\left\lfloor \frac{M}{F}\right\rfloor$ parallel transmissions in each cluster. For instance, with F=3 and M=8, each of the three deployed clusters exploits at least two reception paths on its channel, which explains why FAPM_O is outperforming FAPM. Then, by implementing six and eight channels, Figure 5b shows that FAPM_O gives the same results as FAPM, and no optimization can be made by FAPM_O solution. Besides, since eight channels allow the deployment of eight parallel clusters, the number of EDs that can be supported is bigger than when using six clusters. Moreover, whatever the solution considered, the maximum number of EDs supported increases with the size of the monitoring period.

In Figure 6a, we notice that the number of EDs in the $C_{10,20,20,20,20,10}$ configuration is greater than in the $C_{16,16,16,16,16,16}$ configuration. This is because of the non-uniform distribution of SFs which leads to the absence of some SFs in the sub-clusters. The percentage of EDs with the smallest SF, SF7, and this with the greatest SF, SF12, is half the percentage of EDs with any other SF. This reduces the transmission duration of a representative and by consequence allows the deployment of more sub-clusters in the monitoring period.

The $C_{5,15,30,35,10,5}$ configuration has almost the same behavior as the $C_{10,20,20,20,20,10}$ configuration, because the numbers of EDs with the smallest SF and with the greatest SFs are smaller than for any other SF. The transmission of a representative of 20 EDs is organized into seven sequential sub-clusters, not all filled with *M* EDs with OAPM_D, whereas OAPM_O fills each sub-cluster with *M* EDs which results into three sequential sub-clusters. Since the transmission time needed by three sub-clusters is smaller than for seven sub-clusters, the number of EDs supported by OAPM_O is always bigger than that obtained by OAPM_D which is confirmed by Figure 7a.

**Figure 8:**
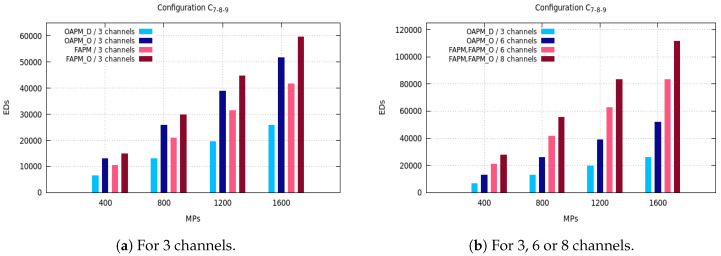
Number of EDs supported by by OAPM_D, OAPM_O, FAPM and FAPM_O in $C_{33,33,33,0,0,0}$.

Figure 8a,b show that with the $C_{33,33,33,0,0,0}$ configuration, the number of EDs supported is greater than in any other configuration. As depicted in Figure 8a, for three channels and a monitoring period of 1600 s, the number of EDs supported by OAPM_D and FAPM is greater than 20,000 and 40,000, respectively. Such numbers are not obtained by any other configuration. And this is due to the lowest transmission time per sub-cluster which is equal to $T_{9}$ in OAPM_D and the sum of $T_{9},T_{8},T_{7}$ in FAPM. Since all SFs are not present, OAPM_O and FAPM_O improve OAPM_D and FAPM, respectively. Figure 8b shows that the number of EDs supported exceeds 50,000 and 80,000 for a monitoring period of 1600 s and six receive paths. For eight channels, that number exceeds 111,000 in both FAPM and FAPM_O solutions with eight receive paths.

**Figure 9:**
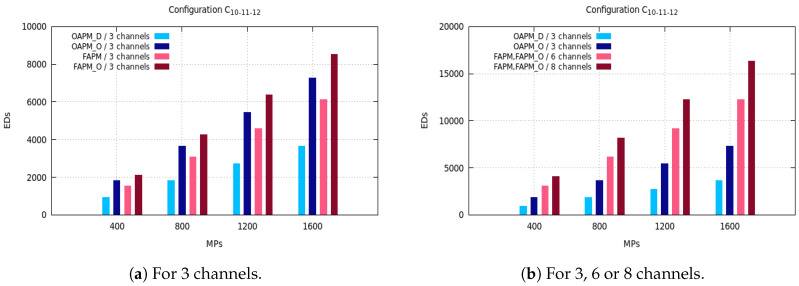
Number of EDs supported by OAPM_D, OAPM_O, FAPM and FAPM_O in $C_{0,0,0,33,33,33}$.

Unlike the previous configuration, Figure 9a,b show that the $C_{0,0,0,33,33,33}$ configuration gives the worst results of our study in terms of number of EDs supported. From Figure 9a, we see that the number of EDs supported by OAPM_D cannot exceed 909 with three channels (three RPs) and a monitoring period of 400 s. When OAPM_O exploits six RPs that number does not exceed 1818. Furthermore, from Figure 9b the number of EDs supported does not exceeds a threshold of 20,000 for FAPM and FAPM_O at their best implementation (8 channels, a monitoring period of 1600 s). Notice that this threshold corresponds to the number of EDs supported by the same solutions with the lowest monitoring period (i.e., 400 s) with six channels in $C_{33,33,33,0,0,0}$ configuration. This is obviously explained by the presence of the longest transmission time per sub-cluster, which is equal to $T_{12}$ in OAPM_D and the sum of $T_{10},T_{11},T_{12}$ in FAPM.

The general conclusions which apply whatever the configuration considered and whatever the solution, are the following. The maximum number of EDs supported increases with the size of the monitoring period. FAPM is always better than OAPM_D, and FAPM_O always outperforms OAPM_O. FAPM and FAPM_O are able to take the best advantage of all the frequency channels to support more EDs. In configurations where all SFs are not present (e.g., $C_{33,33,33,0,0,0}$, $C_{0,0,0,33,33}$) and three frequency channels, OAPM_O supports a greater number of EDs than FAPM.

To summarize the results obtained in Figure 5, Figure 6 and Figure 7, whatever the configuration, uniform or not, whatever the spreading factors present in the monitoring area, whatever the number of frequency channels, whatever the size of the monitoring period, FAPM_O supports the highest number of EDs. It plainly uses the frequency channels available. FAPM outperforms OAPM_D, whereas OAPM_O improves OAPM_D when all SFs are not present.

### 6.2. Simulation Parameters

The simulation is based on the NS3.29 LoRaWAN module [33]. The implemented architecture consists of one gateway and a variable number of end devices deployed in a circular area of 6000 m radius around the GW. The end devices are class A devices. The number of frequency channels of the gateway varies in {3,6,8}.

With the simulation parameters given in Table 5, we evaluate the reliability measured by the Packet Delivery Ratio (PDR) provided by OAPM_D, OAPM_O, FAPM and FAPM_O in the five configurations considered, while progressively increasing the number of End Devices. We also measure the energy consumption of any end device for all the spreading factors used.

### 6.3. Packet Delivery Ratio

**Figure 10:**
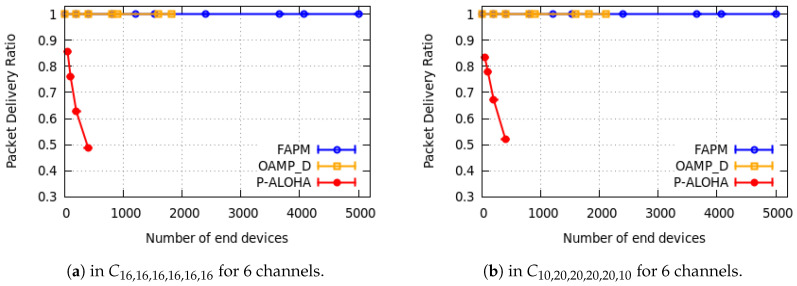
PDR of FAPM, OAPM_D and pure Aloha versus the number of End Devices.

**Figure 11:**
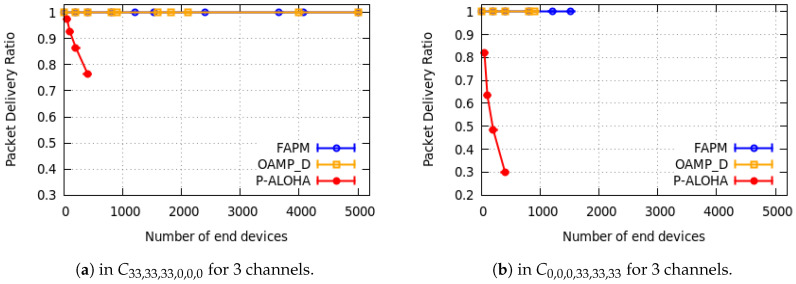
PDR of FAPM, OAPM_D and pure Aloha versus the number of End Devices.

**Figure 12:**
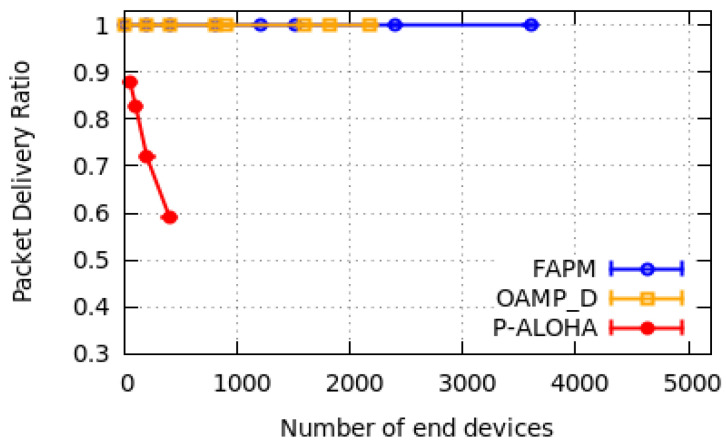
PDR of FAPM, OAPM_D and pure Aloha in $C_{5,15,30,35,10,5}$ with 3 channels versus the number of End Devices.

In this subsection, only some examples of simulation results are reported: one example per configuration. They are depicted in Figure 10, Figure 11 and Figure 12 for a monitoring period equal to 400 s.

We measured the PDR versus the number of EDs devices supported. Due to the hardware limitation, we have considered a maximum of 5000 EDs in our simulation. All curves in Figure 10, Figure 11 and Figure 12 corroborate the theoretical results presented in Section 4.3 and Section 5.4. As shown in Figure 10a,b, the PDR of OAPM_D solution stabilises at 1 up to 1818 and 2020 EDs for the configurations $C_{16,16,16,16,16,16}$ and $C_{10,20,20,20,20,10}$, respectively, which corresponds exactly to the theoretical results given previously. Moreover, Figure 10a,b and Figure 11a show that the PDR of the FAPM solution corroborates theoretical results and stabilises at 1 up to 5000 EDs. In fact, ED numbers greater than 5000 have not been simulated due to hardware limitations. And this restriction is also valid for OAPM_D results presented in Figure 11a. Simulation results illustrated in Figure 11b and Figure 12 are compliant with the theoretical results obtained for each solution. Pure Aloha gives the worst PDR for all the configurations evaluated and for a much smaller number of EDs. This result shows the high efficiency of the solutions proposed.

### 6.4. Energy Consumption

The energy consumed by any ED during a synchronization period is the sum of:

- the energy consumed in transmitting its monitoring report once per monitoring period. Since the monitoring report message is not acknowledged and there is no retransmission, the energy consumed for the transmission in one synchronization period is equal to the Transmission Power, $Power_{Tx}$, times $T_{Rep}$, the transmission duration of a monitoring report using the Spreading Factor of this ED, times the number of monitoring periods in a synchronization period. This energy is the same for all the solutions proposed.
- the energy consumed in listening to the medium, which is equal to the Idle power times the synchronization guard SG. Again, this energy is the same for all the solutions proposed.
- the energy consumed in receiving the synchronization message, which is equal to the Receive Power, $Power_{Rx}$, times the transmission duration of the synchronization message, which is the same for the solutions proposed.

Finally, the energy consumed by any ED per synchronization period is given by:

(25) $$\begin{array}{c} EnergyperSynchroPeriod=nMPperSP\times T_{Rep}\times Power_{Tx}+T_{Sync}\times Power_{Rx}+SG\times Power_{Idle}+\\ \left(SP-nMPperSP\times T_{Rep}-T_{Sync}-SG\right)\times Power_{Sleep}. \end{array}$$

where $T_{Rep}$ is the Transmission Time on Air of the monitoring report by this ED, and $T_{Sync}$ the Transmission Time on Air of the synchronization message (using the maximum SF used in the monitoring area) that the ED has to receive. It is worth noting that this energy is the same for all the solutions proposed.

**Figure 13:**
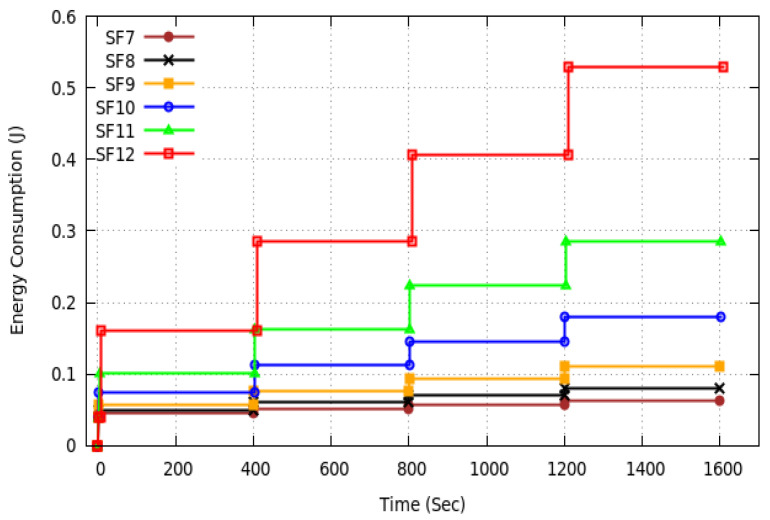
ED Energy Consumption during a Synchronization period.

We evaluate by simulation the energy consumed by any ED in a synchronization period including four monitoring periods. Since this energy depends on the spreading factor used by the ED considered, six EDs are selected: one per spreading factor. Figure 13 depicts their energy consumption measured in the simulations. These results are fully compliant with the theoretical results given by Equation (25). For any spreading factor, the energy consumption curve consists of four steps corresponding to the four monitoring periods in the synchronization period. The horizontal part of each step highlights the almost zero energy consumption when the ED is sleeping. It is worth noting, that EDs far fom the GW use the highest SFs which induce a greater energy consumption.

From Equation (25), we can deduce the lifetime of any ED battery with initial capacity Capacity as follows:

(26) $$Lifetime=\frac{Capacity\times Synchronization\_Period}{EnergyperSynchroPeriod}$$

**Figure 14:**
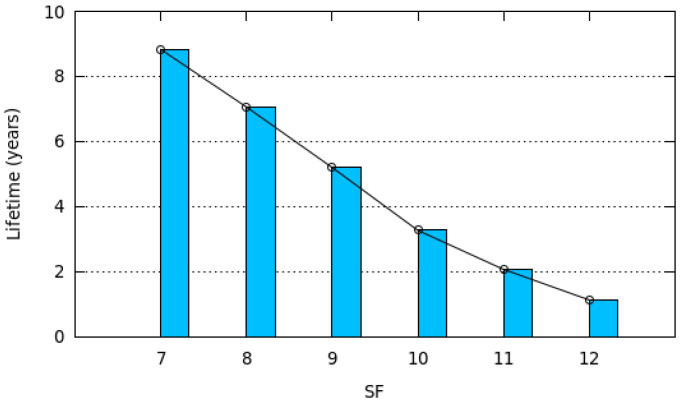
Network lifetime vs Spreading Factor.

Figure 14 depicts the lifetime of any ED equipped with a battery capacity of 1000 mAh. We notice that the ED lifetime decreases when the spreading factor increases. The smaller lifetime of 1.13 year is obtained for SF12, whereas SF7 obtains a network lifetime of 8.83 years.

The duty cycle of any ED is expressed as the percentage of time in a synchronization period during which the ED considered is transmitting its monitoring report or receiving the synchronization message. It is given by:

(27) $$Duty\_cycle\left(ED\right)=\frac{T_{Rep}\times nMPperSP+T_{Sync}+SG}{SP}$$

Hence, the duty cycle of any ED does not depend on the solution studied but only on its transmit power, its receive power, its spreading factor and the spreading factor used to transmit the synchronization message.

Table 6 gives the duty cycle of each ED according to its spreading factor for a monitoring period of 400 s and a synchronization period of 1602 s. It is worth noting that all EDs meet the requirement of a duty cycle ≤1%.

## 7. Applicability of These Solutions

In the previous section, we have compared the scalability of OAPM_D, OAPM_O, FAPM and FAPM_O expressed by the maximum number of EDs supported. We have also evaluated the energy consumption of each solution. In this section, we discuss the applicability of the solutions proposed with regard to the requirements expressed by IoT applications. These constraints involve:

- the number of EDs deployed.
- the geographical coordinates of each ED. The spreading factor used by the ED considered is deduced from its distance to the GW. The cluster to which this ED belongs is computed from its geographical coordinates.
- the average delay between two consecutive application messages generated by the same ED, also called inter-arrival time, and the standard deviation.
- the message size.
- the maximum acceptable data latency, which is defined as the maximum time elapsed between the generation of the message including these data and its receipt by the GW.
- the reliability required, which is expressed by the PDR.
- the minimum lifetime of ED, which is requested by the application.
- the maximum duty cycle of ED, which is accepted by the application.
- the existence of an urgent traffic. If it exists, it should be described as the normal traffic is and the reliability and latency constraints should be expressed.
- the ED cost. In this paper, we assume that the ED cost is proportional to the complexity of the solution implemented in this ED.
- The coexistence with other applications sharing this GW.

### 7.1. Complexity

In all the solutions presented, there is no complexity in the EDs: each ED has only to transmit its monitoring report at the time and on the frequency channel assigned by the Network Server. All the complexity is left to the Network Server for the configuration of End Devices and to the GW for receiving simultaneous messages sent by different EDs during the monitoring phase.

### 7.2. Data Gathering Duration

The maximum number of EDs supported by any solution presented is computed from the monitoring period to ensure collision-free transmissions of all EDs. Hence, the data gathering duration is very close to the Monitoring period, when the number of EDs is maximum. Notice that this maximum depends on the solution considered.

If the number of EDs is small with regard to the maximum, the GW may support several monitoring applications. In such a case, the solution minimizing the data gathering duration such as FAPM_H presented in the next section, will be preferred to make the coexistence of several applications easier. Another advantage of a solution minimizing the data gathering duration is to ensure a better time consistency of all the samples collected since they have been collected at closer times.

### 7.3. Data Latency

Since in all the solutions proposed, the transmission of the monitoring report produced by each ED is scheduled, the generation of a new message corresponding to a new measure is not immediately followed by its transmission, some latency may exist.

In the worst case, the new message is generated just after the ED considered starts the transmission of its monitoring report. Consequently, the new message will be transmitted in the next monitoring period. In the worst case, this next monitoring period is preceded by a synchronization. The maximum latency is equal to:

(28) $$MaxLatency=MP+T_{Sync}+2SG+T_{Rep},$$

where $T_{Rep}$ denotes the Time on Air of the monitoring report transmitted by this ED, $T_{Sync}$ is the Time on Air of the synchronization message transmitted with the highest SF in the configuration.

Since a new message may be generated at any time in the monitoring period and the next monitoring period is preceded by a synchronization once per nMPperSP, the average latency is equal to:

(29) $$AverageLatency=\frac{MP}{2}+\frac{T_{Sync}+2SG}{2nMPperSP}+T_{Rep}.$$

### 7.4. Urgent Traffic Support

Some monitoring IoT applications support two classes of traffic: urgent and normal. Urgent traffic should be delivered promptly to the GW. If the requirements for normal traffic are fully compliant with the average latency and the maximum latency given by Equation (29) and by Equation (28), respectively, it is usually not the case for the urgent traffic. The solutions proposed can easily be extended by assuming that only a small number of EDs can generate urgent messages (Assumption A15). These EDs, denoted Urg_EDs, maintain two buffers: one for an urgent message and one for a normal message, whereas the remaining EDs, denoted Norm_EDs, maintain only one buffer for a normal message. The monitoring period is subdivided into a multiple of urgent monitoring periods. In each urgent monitoring period, there are three parts:

- The first one is left to Urg_EDs. They are the only devices allowed to transmit in this part. They transmit an urgent message, if it exists, and a normal message otherwise. As a consequence, an Urg_ED has as many opportunities to transmit urgent messages as the number of urgent monitoring periods in a monitoring period.
- The second part is left to some Norm_EDs. Norm_EDs are assigned to urgent monitoring periods in such a way that each Norm_ED has a single opportunity to transmit in each monitoring period.
- This third part may be empty, it depends on the application requirements. In the third part, all devices are sleeping to save energy.

With Assumption A15, the maximum latency of urgent traffic is given by:

(30) $$MaxUrgentLatency=UMP+T_{Sync}+2SG+T_{Urg},$$

where UMP is the Urgent Monitoring Period, $T_{Urg}$ denotes the Time on Air of the urgent message transmitted by this ED.

With Assumption A15, the average latency of urgent traffic is equal to:

(31) $$AverageUrgentLatency=\frac{UMP}{2}+\frac{T_{Sync}+2SG}{nMPperSP}+T_{Urg}.$$

If Assumption A15 is not met, the monitoring period becomes very close to the urgent monitoring period and the number of EDs supported decreases to meet the latency of urgent messages. If one requirement of the IoT application is not met, the solution cannot be applied.

### 7.5. Probabilistic Traffic

Up to now we have only considered the case where each ED generates a single message in each monitoring period. We now take into account the fact any ED may generate 0, 1 or more messages per monitoring period. Traffic generation is now probabilistic. For simplicity reasons, the size of the buffer of each ED is set to one monitoring message. When a new message is generated, it replaces the previous one. This is consistent with the data freshness required by the monitoring applications. As a consequence, a given monitoring message may be never transmitted if a new message has already replaced it.

**Figure 15:**
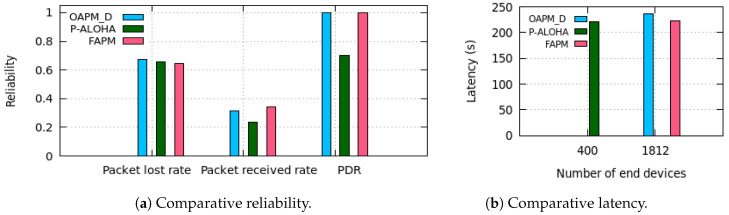
Comparative performance evaluation of pure Aloha, OAPM_D and FAPM in $C_{16,16,16,16,16,16}$.

We now consider probabilistic traffic. Each ED generates a Poisson traffic with inter-arrival time equal to the monitoring period MP=400 s. We consider the uniform configuration with six channels and compare the performances of the solutions studied in terms of reliability in Figure 15a and latency of the messages delivered to the GW in Figure 15b, respectively. The number of EDs for each solution evaluated is equal to 1812, except for Aloha where it is equal to 400. With regard to latency, simulation results corroborate the theoretical results. With regard to reliability, simulation results provide the rates of (i) messages overwritten (i.e., denoted lost in Figure 15a), and messages received over the total number of messages generated, and (ii) messages delivered among those transmitted, which is the PDR. It appears that the main reason for unreliability is due to the overwriting of messages in the case of a buffer of size one message. To reduce these losses, a smaller monitoring period could be used. All the solutions proposed ensure a PDR equal to one, whereas pure Aloha with a smaller number of EDs (i.e., less than the quarter of the number of EDs used for the other solutions) ensures a PDR equal to 0.7. This shows the efficiency of the solutions proposed. Figure 15b shows an average latency around 222 s for all the solutions evaluated, in accordance with Equation (29).

CSMA/CA and pure Aloha have been well analyzed and evaluated in the literature. However their performances have been compared considering saturated traffic on all devices. This assumption is no longer valid for IoT monitoring applications. In addition, the metric evaluated was the network throughput, whereas in IoT monitoring applications, metrics such as PDR, latency and energy consumption are more relevant. In [34], the authors show by simulation that to ensure a PDR ≥0.7 with a transmission opportunity of $165\times 10^{-5}$ corresponding for instance to the transmission of a message of 20 bytes with SF = 11, pure Aloha requires a number of EDs ≤220, wheras CSMA/CA allows up to 500 EDs. The reason is that CSMA/CA senses the medium before transmitting and differs the transmission if the medium is busy. This behavior helps to reduce the number of collisions. However, this improvement is obtained at the expense of a greater duty cycle.

Both simulation results and theoretical results obtained in [35] via a Markov model demonstrate that to provide a PDR ≥0.7 in the uniform configuration $C_{16,16,16,16,16,16}$ in a LoRaWAN network of 300 EDs, pure Aloha requires a message generation rate ≤0.002, which is less than one message per monitoring period of 400 s, whereas CSMA/CA supports a message generation rate up to 0.004, which is the double. Furthermore, the authors prove that the coexistence of pure Aloha and CSMA/CA devices in the same LoRaWAN network benefits to pure Aloha devices.

As a conclusion, CSMA/CA outperforms pure Aloha in terms of PDR, but is unable to support the same number of EDs as OAPM_D and FAPM, while maintaining a PDR ≥0.7.

## 8. A Hybrid Solution with FAPM_H

The intuitive idea behind FAPM_H is to use the GW at the maximum of its possibilities: simultaneous demodulation of *M* messages. To achieve this goal, FAPM and OAPM_D are used jointly: clusters transmit in parallel as in FAPM and some End Devices transmit in parallel as in OAPM_D. The sub-clusters are designed in such a way that the number of messages simultaneously transmitted to the GW is as close to *M* as possible, but without exceeding it. The improved performance is obtained at the cost of a higher complexity in the scheduling of End Device transmissions, which is the solution of the following optimization problem.

### 8.1. FAPM_H Solution of An Optimization Problem

Let τ denote the time unit used to express all the transmission durations and the monitoring guard MG as multiples of τ. For the sake of simplicity, we include the monitoring guard into the transmission time of any monitoring report. As a consequence, in the following $T_{i}$ denotes the number of time units necessary to transmit the monitoring report of any ED using $SF_{i}$ plus the monitoring guard.

Without loss of generality, we reason on discrete time and abusively call the $t^{th}$ time unit, slot t,∀t. It is worth noting that these slots are purely virtual and have no existence on the medium. Let $X_{c,r}^{i}\left(t\right)$ be the decision variable that is equal to 1 if and only if ED *i* is transmitting in slot *t* on receive path *r* assigned to channel *c* with a spreading factor equal to $SF_{i}$, and 0 otherwise.

**The FAPM_H optimization problem can be written as follows:**

**Goal:**

- Minimize the transmission duration on any receive path assigned to any frequency channel:
  (32) $$\min\;\max_{Channel\;c}\;\max_{RP\;r}\;\sum_{ED\;i}\;\sum_{time\;t}X_{c,r}^{i}\left(t\right)$$

**With the following constraints:**

1. At any time *t*, any transmission not finished at the end of slot *t* is going on in slot t+1 on the same receive path and the same channel:
  (33) $$\begin{array}{c} \mathrm{If}\;X_{c,r}^{i}\left(t\right)=1\;\mathrm{and}\;\sum_{s=t}^{t-T_{i}+1}X_{c,r}^{i}\left(s\right)<T_{i},\;\mathrm{then}\;\\ X_{c,r}^{i}\left(t+1\right)=1,\forall \;\mathrm{time}\;t,\forall \;\mathrm{channel}\;c,\forall \;\mathrm{receive}\;\mathrm{path}\;r\;\mathrm{assigned}\;\mathrm{to}\;c,\forall \;ED\;i. \end{array}$$
2. At any time *t*, there are no more than *F* frequency channels used:
  (34) $$\sum_{Channel\;c}\;\max_{RP\;r}\;\sum_{ED\;i}X_{c,r}^{i}\left(t\right)\leq F,\forall \;\mathrm{time}\;t.$$
3. At any time *t*, at most *M* messages are simultaneously decoded by the GW:
  (35) $$\sum_{Channel\;c}\sum_{RP\;r}\sum_{ED\;i}X_{c,r}^{i}\left(t\right)\leq M,\forall \;\mathrm{time}\;t.$$
4. At any time, at most 6 messages are simultaneously received by the GW on the same frequency.
  (36) $$\sum_{RP\;r\;of\;channel\;c}\sum_{ED\;i}X_{c,r}^{i}\leq 6,\forall \;\mathrm{time}\;t,\forall \;\mathrm{channel}\;c.$$
5. At any time, there are no two EDs that simultaneously transmit with the same SF and on the same frequency:
  (37) $$\sum_{RP\;r\;of\;channel\;c}\sum_{ED\;k\neq i\;with\;SF_{k}=SF_{i}}X_{c,r}^{k}\left(t\right)\leq 1,\forall \;\mathrm{time}\;t,\forall \;\mathrm{channel}\;c,\forall \;ED\;i\;\mathrm{transmitting}\;\mathrm{on}\;\mathrm{channel}\;c.$$

We now apply FAPM_H to two configurations: the first one, $C_{16,16,16,16,16,16}$, is uniform whereas the second one, $C_{5,15,35,30,10,5}$, is not.

### 8.2. FAPM_H for the Uniform Configuration

For the uniform configuration with F=3 frequencies and M=8 receive paths, FAPM_H schedules the EDs as follows:
Freq 1Receive Path 1SF12SF9SF9SF8SF7SF7
Receive Path 2SF11SF11SF8SF11


Receive Path 3SF10SF10SF12


Freq 2Receive Path 4SF12SF9SF9SF8SF7SF7
Receive Path 5SF11SF11SF8SF11


Receive Path 6SF10SF10SF12


Freq 3Receive Path 7SF12SF9SF9SF8SF7SF7
Receive Path 8SF10SF10SF12SF8

Total Time
$3T_{11}$ +$T_{8}$






It is worth noting that with this schedule, all the available frequencies and all the available receive paths are used. In addition, the total transmission times on each receive path are close. This explains why FAPM_H outperforms FAPM_O, as shown hereafter: The maximum number of EDs supported by FAPM_H is given by:

(38) $$ED_{FAPMH3freq}=3\times 2\times 6\times \left\lfloor \frac{MP}{3T_{11}+T_{8}+4MG}\right\rfloor .$$

For instance, for a monitoring period of 400 s, FAPM_H is able to support 6876 EDs, whereas FAPM_O supports only 3996 EDs, leading to an improvement of 72%. The data gathering duration with FAPM_H is equal to $3T_{11}+T_{8}+3MG=$ 2089 ms, whereas it is equal to $2(T_{12}+T_{10}+T_{8})+5MG=3596$ ms, which represents a decrease of 42%.

With F=8 frequencies and M=8 receive paths, FAPM_H uses the eight frequencies and the eight receive paths, one receive path per frequency, to schedule six EDs sequentially on each frequency. FAPM_H has exactly the same behavior as FAPM_O and FAPM. Here again, all available frequencies and all available receive paths are used and are busy for exactly the same duration. The maximum number of EDs supported by FAPM_H is given by:

(39) $$ED_{FAPMH8freq}=8\times 6\times \left\lfloor \frac{MP}{T_{12}+T_{11}+T_{10}+T_{9}+T_{8}+T_{7}+6MG}\right\rfloor .$$

For instance, for a monitoring period of 400 s, FAPM_H is able to support the same number of EDs as FAPM_O, namely 7056.

### 8.3. FAPM_H for the $C_{5,15,35,30,10,5}$ Configuration

For the $C_{5,15,35,30,10,5}$ configuration with F=3 frequencies and M=8 receive paths, FAPM_H schedules the EDs as follows:
Freq 1Receive Path 1SF12SF11SF9SF8SF8SF8SF7




Receive Path 2SF11SF10SF9SF9SF10SF10SF10



Freq 2Receive Path 3SF12SF11SF8SF8SF8






Receive Path 4SF11SF9SF9SF9SF9SF9SF9SF9SF9SF9SF7
Receive Path 5SF10SF10SF10SF10SF10SF10SF10



Freq 3Receive Path 6SF12SF11SF8SF8SF8






Receive Path 7SF11SF9SF9SF9SF9SF9SF9SF9SF9SF9SF7
Receive Path 8SF10SF10SF10SF10SF10SF10SF10



Time
$7T_{10}$








This schedule shows that all available frequencies and all receive paths are used for similar durations. This is the main reason why FAPM_H outperforms FAPM_O. The maximum number of EDs supported by FAPM_H is given by:

(40) $$ED_{FAPMH3freq}=3\times 20\times \left\lfloor \frac{MP}{7T_{10}+7MG}\right\rfloor .$$

For instance, for a monitoring period of 400 s, FAPM_H is able to support 9180 EDs, whereas FAPM_O supports only 5520 EDs, leading to an increase of 66%. The data gathering duration with FAPM_H is equal to $7T_{10}+6MG=2607$ ms, whereas it is equal to 4323 ms with FAPM_O, which represents a decrease of 39.7%.

With F=8 frequencies and M=8 receive paths, FAPM_H uses the eight frequencies and the eight receive paths, one receive path per frequency, to schedule 20 EDs sequentially on each frequency. FAPM_H has exactly the same behavior as FAPM_O and FAPM. Here again, all available frequencies and all available receive paths are used and are busy for exactly the same duration. The maximum number of EDs supported by FAPM_H is given by:

(41) $$ED_{FAPMH8freq}=20F\times \left\lfloor \frac{MP}{T_{12}+2\times T_{11}+6\times T_{10}+7\times T_{9}+3\times T_{8}+T_{7}+20MG}\right\rfloor .$$

For instance, for a monitoring period of 400 s, FAPM_H is able to support the same number of EDs as FAPM_O, namely 9600.

### 8.4. Conclusion on FAPM_H and the Other Solutions Studied

From these results, we can conclude that:

- When M=F, FAPM, FAPM_O and FAPM_H which behave exactly the same, are optimal.
- When F>M, only *M* frequencies can be used simultaneously. In such a case, FAPM, FAPM_O and FAPM_H which behave exactly the same, are optimal for this value of *M*.
- When F<M, FAPM_H provides better results by combining inter-channel parallelism (i.e., several frequency channels) and intra-channel parallelism (i.e., several receive paths per channel) while trying to balance the total transmission duration on each receive path, which leads to a smaller data gathering duration.

**Figure 16:**
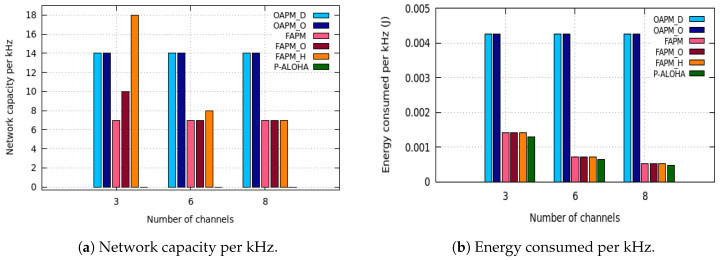
Comparison of Pure Aloha, the OAPM family and the FAPM family.

**Figure 17:**
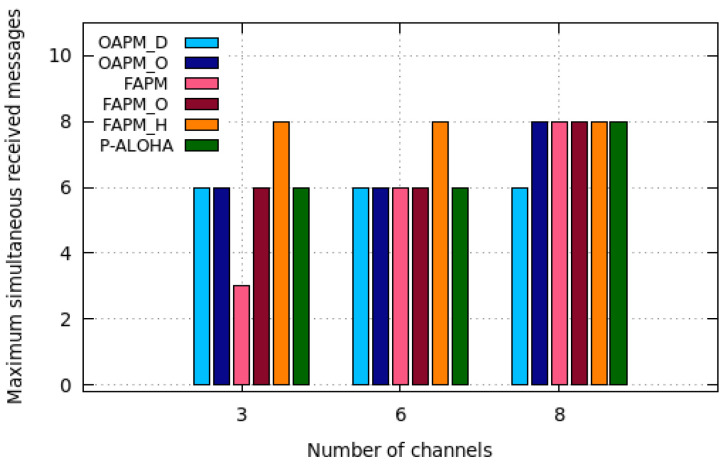
Maximum number of simultaneous messages received by the GW for Pure Aloha, the OAPM family and the FAPM family.

Since the solutions proposed may use different numbers of channels, they are compared in terms of network capacity per kHertz and energy consumption per kHertz. Figure 16a,b and Figure 17, depict the network capacity per kHz, the energy consumption per kHz and the maximum number of messages simultaneously received by the GW for pure Aloha, the OAPM family and the FAPM family, with as an example the $C_{16,16,16,16,16,16}$ configuration, a monitoring period of 400 s, a deterministic traffic and a number of frequency channels ranging from 3 to 8.

For pure Aloha, the maximum number of EDs supported is computed to ensure a Packet Delivery Ratio (PDR) higher than or equal to 70%. With regard to the energy consumed, Pure Aloha does not require the synchronization of EDs. This explains why it consumes less energy than the solutions proposed. Notice however that this functionality is very often required by the IoT applications in order to order events occurring throughout the monitoring area and detect causality between events.

However, these figures may be misleading, because multiplying the capacity per kHz by the number of kHz made available for LoRaWAN does not give the real network capacity. This is due to the limitation introduced by *M*, the maximum number of simultaneous messages the GW is able to receive. Figure 17 depicts the maximum number of simultaneous messages received by the GW for Pure Aloha, the OAPM family and the FAPM family, for a value of M=8. It follows that FAPM_H uses all the resources available both in terms of channels and simultaneous messages received by the GW.

## 9. Conclusions

Since the LoRaWAN network allows long-range and robust communication while reducing the energy consumption, it appears like an excellent candidate to support an ever increasing number of IoT monitoring applications. To avoid poor reliability when a large number of End Devices (EDs) are deployed, we propose two families of solutions ensuring collision-free transmissions. In the OAPM family which is TDMA-based, all clusters transmit in sequence, whereas up to six EDs with different spreading factors belonging to the same cluster are allowed to transmit in parallel. OAPM_O is an optimized variant of OAPM_D using several frequency channels per cluster to allow several EDs with the same spreading factor to transmit in parallel. In the FAPM family which is FDMA-based, all clusters transmit in parallel, each cluster on its own frequency. Within each cluster, all EDs transmit in sequence in FAPM. FAPM_O is an optimized variant of FAPM allowing several EDs of the same cluster to transmit in parallel. These solutions have been evaluated theoretically and by simulation with NS3 in various configurations, uniform or not, with some or all spreading factors present in the monitoring area, different numbers of frequency channels, and different sizes of the monitoring period. The simulation results show that all these solutions ensure that no messages are lost due to collisions. They corroborate the theoretical results of the maximum number of EDs supported by each solution: FAPM_O supports the highest number of EDs, because of its better use of the frequency channels available. FAPM outperforms OAPM_D, whereas OAPM_O improves OAPM_D when all SFs are not present. Finally, a hybrid solution, FAPM_H, is proposed. It combines FAPM and OAPM to achieve both inter-channel parallelism and intra-channel parallelism while minimizing the data gathering duration. This is done by maximizing the parallelism of transmissions under the constraints given by the maximum number of available frequency channels and the maximum number of messages the gateway is able to demodulate simultaneously. The complexity of solving this optimization problem is left to the Network Server. As a further work, the evaluation of FAPM_H and its comparison with other schemes will be carried on. The proposed solutions will be implemented on real testbeds. They will also be tested in different use case applications to confirm their effectiveness. For applications requiring a high reliability, application messages can be acknowledged. However, the impact of acknowledgments on the duty cycle will strongly limit the capacity of LoRaWAN. More generally, the coexistence of several IoT applications with heterogeneous requirements in the same LoRaWAN gateway is a challenging study. 

## Figures and Tables

**Table 1:** LoRaWAN default channels and duty cycle limitations.

Channel	Central Frequency (MHz)	Duty Cycle	Regulatory Regime	Max Effective Radiated Power (ERP)
1	868.1			
2	868.3	1%	h 1.5	14 dBm
3	868.5			
4	868.85	0.1%	h 1.6	14 dBm
5	869.05			
6	869.525	10%	h 1.7	27 dBm

**Table 2:** Notations.

MA	Monitoring Application
GW	Gateway
ED	End Device
NS	Network Sever
*F*	Number of frequency channels the GW simultaneously listens to, F=3, 6 or 8
*M*	Maximum number of messages simultaneously demodulated by the GW, also called number of receive paths of the GW, M=8
SP	Synchronization Period
MP	Monitoring Period
TW	Time Window
TT	Transmission Time
Δ	The clock of any ED is synchronized within Δ to the GW clock
SG	Synchronization guard time between a downlink synchronization message followed by an uplink monitoring message, or vice-versa
MG	Monitoring guard time between two uplink monitoring messages
SF	Spreading Factor, SF∈{7,8,9,10,11,12}
$T_{SF}$	Transmission Time of a monitoring message using the spreading factor SF
$ED_{OAPMD}$	Maximum number of EDs supported by OAPM_D
$ED_{FAPM}$	Maximum number of EDs supported by FAPM

**Table 3:** Sub-cluster versus number of frequency channels for OAPM_O.

Channels	Sub-Clusters	Members	Transmission Duration
3, 6 or 8	1	SF7, SF8, SF9, SF10, SF11, SF12, SF9, SF10	$T_{12}+MG$
	2	SF8, SF9, SF10, SF11, SF9, SF10, SF9, SF10	$T_{11}+MG$
	3	SF8, SF9, SF10, SF9	$T_{10}+MG$

**Table 4:** Differences between OAPM_D and FAPM.

	OAPM_D	OAPM_O	FAPM	FAPM_O
	TDMA Based	TDMA Based	FDMA Based	FDMA Based
Channel	Only one channel is used: the same for all clusters	Several channels can be used by a same sub-cluster	One channel per cluster, at most *M* channels	One channel per cluster, at most *M* channels
Receive Path (RP) per channel	Up to six RPs (one per SF) in a same sub-cluster	Several RPs per channel in a same sub-cluster, total # of RPs ≤M	One RP per channel in each cluster	Several RPs per channel, total # of RPs ≤M, a single channel per cluster
Clusters	Yes	Yes	Yes	Yes
Sub-cluster	EDs with ≠ SFs	EDs with possible = SFs but ≠ channels	No sub-cluster	EDs with ≠ SFs
Transmissions of different clusters	Sequential	Sequential	Parallel	Parallel
Transmissions of sub-clusters in a same cluster	Sequential	Sequential	No sub-cluster	Sequential
Transmissions of EDs in a same sub-cluster	Up to min(# of RPs assigned to this sub-cluster, # of ≠ SFs in the sub-cluster) transmissions in parallel	Up to min(# of RPs assigned to this sub-cluster, # of ≠ SFs in the sub-cluster × # of Freq assigned to this sub-cluster) transmissions in parallel	Only one transmission	Up to min(# of RPs assigned to this sub-cluster, # of ≠ SFs in the sub-cluster) transmissions in parallel

**Table 5:** Simulation Parameters.

Parameter	Value
Bandwidth	125 KHz
Channels	Default Channels 868.1, 868.2, 868.3 MHz
Spreading Factors	7, 8, 9, 10, 11, 12
Battery capacity	1000 mAh
TX Power	14 dBm corresponding to $Power_{Tx}=28$ mA
RX power	$Power_{Rx}=11.2$ mA
Idle power	$Power_{Idle}=1.4$ mA
Sleep power	$Power_{Sleep}=15$μA
Supply Voltage	3.3 V
Number of EDs	[0…5000]
Area radius	6000 m
Data payload	21 bytes
Uplink message Type	Unconfirmed
Monitoring Period	400 s, 800 s, 1200 s, 1600 s
Synchronization Period	1602 s
Simulation Time	32,000 s

**Table 6:** Duty_cycle(ED) for a monitoring period of 400 s and a synchronization period of 1602 s.

Spreading Factor	ED Duty_cycle (%)
SF7	0.08
SF8	0.09
SF9	0.11
SF10	0.15
SF11	0.23
SF12	0.39
